# Effects of a ketogenic diet on motor function and motor unit number estimation in aged C57BL/6 mice

**DOI:** 10.1016/j.jnha.2024.100219

**Published:** 2024-04-05

**Authors:** Carlos J. Padilla, Hallie Harris, Jeff S. Volek, Brian C. Clark, W. David Arnold

**Affiliations:** a Department of Kinesiology, University of Wisconsin – Madison, Madison, WI, USA; b Department of Plastic and Reconstructive Surgery, The Ohio State University, Columbus, OH, USA; c Department of Human Sciences, The Ohio State University, Columbus, OH, USA; d Department of Biomedical Sciences, Ohio University, Athens, OH, USA; e Ohio Musculoskeletal and Neurological Institute (OMNI), Ohio University, Athens, OH, USA; f University of Missouri, School of Medicine, Columbia, MO, USA; g NextGen Precision Health Initiative, University of Missouri System, Columbia, MO, USA

**Keywords:** Ketogenic diet, Motor function, Motor units number estimation, Aging

## Abstract

**Objective:**

Pathological, age-related loss of muscle function, commonly referred to as sarcopenia, contributes to loss of mobility, impaired independence, as well as increased risk of adverse health events. Sarcopenia has been attributed to changes in both neural and muscular integrity during aging. Current treatment options are primarily limited to exercise and dietary protein fortification, but the therapeutic impact of these approaches are often inadequate. Prior work has suggested that a ketogenic diet (KD) might improve healthspan and lifespan in aging mice. Thus, we sought to investigate the effects of a KD on neuromuscular indices of sarcopenia in aged C57BL/6 mice.

**Design:**

A randomized, controlled pre-clinical experiment consisting of longitudinal assessments performed starting at 22-months of age (baseline) as well as 2, 6 and 10 weeks after the start of a KD vs. regular chow intervention.

**Setting:**

Preclinical laboratory study.

**Sample size:**

Thirty-six 22-month-old mice were randomized into 2 dietary groups: KD [n = 22 (13 female and 9 male)], and regular chow [n = 15 (7 female and 8 male)].

**Measurements:**

Measures included body mass, hindlimb and all limb grip strength, rotarod for motor performance, plantarflexion muscle contractility, motor unit number estimations (MUNE), and repetitive nerve stimulation (RNS) as an index of neuromuscular junction transmission efficacy recorded from the gastrocnemius muscle. At end point, muscle wet weight and blood samples were collected to assess blood beta-hydroxybutyrate levels.

**Statistical analysis:**

Primary analyses were two-way mixed effects ANOVA (diet and time × diet) to determine the effect of a KD on indices of motor function (grip, rotarod) and indices of motor unit (MUNE) and muscle (contractility) function.

**Results:**

Beta-hydroxybutyrate (BHB) was significantly higher at 10 weeks in mice on a KD vs control group (0.83 ± 0.44 mmol/l versus 0.42 ± 0.21 mmol/l, η^2^ = 0.265, unpaired t-test, p = 0.0060). Mice on the KD intervention demonstrated significantly increased hindlimb grip strength (diet, p = 0.0001; time × diet, p = 0.0030), all limb grip strength (diet, p = 0.0005; time × diet, p = 0.0523), and rotarod latency to fall (diet, p = 0.0126; time × diet, p = 0.0021). Mice treated with the KD intervention also demonstrated increased MUNE (diet, p = 0.0465; time × diet, p = 0.0064), but no difference in muscle contractility (diet, p = 0.5248; time × diet, p = 0.5836) or RNS (diet, p = 0.3562; time × diet, p = 0.9871).

**Conclusion:**

KD intervention improved neuromuscular and motor function in aged mice. This pre-clinical work suggests that further research is needed to assess the efficacy and physiological effects of a KD on indices of sarcopenia.

## 1 Introduction

Muscle function in older adults is critical for maintaining independence in daily life, and impaired physical function is associated with adverse health outcomes and increased risk of mortality [1]. Age-related decline of muscle function has been attributed to changes in both the nervous and muscular systems [2]. Prior work has indicated that there is progressive decline in muscle function (e.g., strength) beginning around 30 years of age with an accelerated loss after 60 years of age [3,4]. The age-related reduction in muscle strength is notably greater than the loss of muscle mass [5], which indicates that other changes in the neuromuscular system beyond atrophy are mechanistically associated with the loss of muscle function [6,7]. As such, it is not surprising that poor motor nerve function is linked to muscle weakness and mobility disability [[8], [9], [10], [11]].

Resistance exercise is the mainstay treatment and prevention strategy for sarcopenia [12]. However, most older adults do not perform resistance exercise training [13], and even among those that do there is a large degree of between person variability in the strength response observed [14,15]. Optimized nutrition has been suggested by many to offer potential benefits for aging [[16], [17], [18], [19], [20], [21]], and studies have suggested that a ketogenic diet (KD) may improve muscle performance and maintenance in the context of both sarcopenia [22] and cachexia [23]. The KD is characterized by high fat intake (60–90%), modest protein intake (10–20%), and very low carbohydrate intake (5–10%, usually <50 g/day) of the total energy content of the diet. From a physiological perspective, the KD results in an increase in plasma ketone bodies, especially β-hydroxybutyrate (BHB), the most abundant ketone found in the circulation [24]. Neuroketotherapy represents a class of bioenergetic medicine therapies that are characterized by the induction of ketosis [25]. The KD has shown protective effects in a broad spectrum of neurological disorders, including models of neurodegeneration, neurogenetic conditions, cerebrovascular disease, and immune-mediated demyelination [[26], [27], [28]].

Accordingly, in the current study, we sought to investigate the effects of a KD on neuromuscular indices of sarcopenia in aged C57BL/6 mice. Specifically, in building on findings that suggest that the KD has positive effects on both muscles and nerves [29,30], we were interested in determining the effects and consequences of a KD on motor unit, neuromuscular junction, and muscle contractile function.

## 2 Methods

### 2.1 Experimental overview

**Fig. 1:**
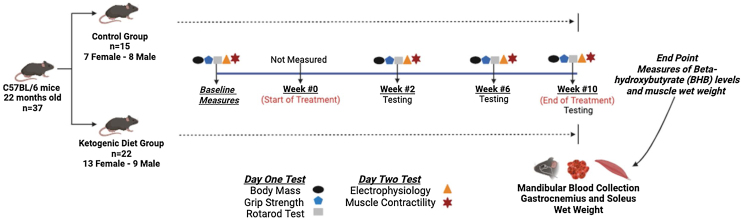
Study design. Thirty-six 22-month-old mice were randomized into 2 groups: a group fed a ketogenic diet ad libitum for 10 weeks (Envigo Teklad TD.96355; 9.2% protein, 0.3% carbohydrates and 90.5% fat) [n = 22 (13 female and 9 male)] and a group fed regular chow ad libitum for 10 weeks (Envigo Teklad 7012; 25% protein, 58% carbohydrates and 17% fat) [n = 15 (7 female and 8 male)]. Body mass, electrophysiology, muscle contractility, grip strength, and motor performance were measured at baseline as well as after 2, 6, and 10 weeks of the intervention. All electrophysiology and contractile measurements were done on the right leg. At endpoint (week 10), ketone blood levels (Beta – Hydroxybutyrate, mmol/l) were assessed using blood obtained by a mandibular bleed and gastrocnemius and soleus muscle wet weights.

All procedures were approved and performed in accordance with the Institutional Animal Care and Use Committee of The Ohio State University. C57BL/6 mice were obtained from the National Institute on Aging colony at 21 months of age. They acclimatized for 2 weeks with regular food and then began the study at 22 months. Mice were kept on a 12 -h light-dark cycle with free access to food and water. Also, they were evaluated at the beginning of the study and then randomly assigned to a KD diet (n = 22, 13 females and 9 males) or regular food (n = 15, 7 females and 8 males) ad libitum. Longitudinal assessments were performed at baseline as well as 2, 6 and 10 weeks after the start of the respective dietary intervention. Outcome measures included body mass, hindlimb and all limb grip strength, rotarod latency to fall to assess motor performance, and electrically stimulated plantarflexion muscle contractility. Additionally, in the gastrocnemius muscle of the right leg we used electrophysiological techniques to assess compound muscle action potential (CMAP) amplitude, and incremental stimulation for motor unit number estimates (MUNE), which assesses the number of functioning motor units [31], as well as repetitive nerve stimulation at 50 Hz to assess neuromuscular junction transmission [32]. At the end point (week 10 post intervention), blood samples were collected (submandibular bleed) to assess blood ketone levels (Beta-hydroxybutyrate-BHB). The study design is presented in Fig. 1.

### 2.2 Diet intervention

We implemented a daily monitoring routine during the 10-week study to ensure the consistent availability of food. Every morning, as part of our standard care procedures, we thoroughly checked each cage to confirm that there was a sufficient amount of food available for the day. This practice was strictly followed to maintain the health and well-being of all animals throughout the duration of the study. Our commitment to providing uninterrupted access to food aligns with both the ethical standards of animal care and the scientific integrity of the study. We ensured that the dietary needs of the animals were met continuously, thereby preventing any confounding factors that could arise from unintended fasting or food deprivation. By detailing this aspect of our animal care protocol, we aim to reassure that the welfare of the animals was a top priority, and that consistent food availability was maintained to support the validity of our research findings. Diets were prepared by Envigo Teklad Diets (Madison, WI) and placed in a small container inside the mouse cages. The High-Fat Ketogenic Teklad Custom Diet (TD.96355) used a fat to protein + carbohydrate ratio. For the regular chow, the 7012 Teklad LM-485 Mouse Sterilizable Diet was used, which is a fixed formula, autoclavable diet, manufactured with high quality ingredients and designed to support the growth of rodents. Typical concentrations of isoflavones (daidzein + genistein aglycone equivalents) range from 300 to 600 mg/kg. 7012 is supplemented with additional vitamins to ensure nutritional adequacy after autoclaving. The ingredients used were: Ground corn, dehulled soybean meal, ground oats, wheat middling, dehydrated alfalfa meal, soybean oil, corn gluten meal, calcium carbonate, dicalcium phosphate, brewers dried yeast, iodized salt, choline chloride, kaolin, magnesium oxide, L-lysine, DL-methionine, ferrous sulfate, menadione sodium bisulfite complex (source of vitamin K activity), vitamin E acetate, thiamin mononitrate, calcium pantothenate, manganous oxide, niacin, copper sulfate, zinc oxide, vitamin A acetate, pyridoxine hydrochloride, riboflavin, vitamin D3 supplement, vitamin B12 supplement, folic acid, biotin, calcium iodate, and cobalt carbonate. Nutrient information and values for the ketogenic diet and regular chow were selected and calculated from ingredient analysis and manufacturer data. The dietary information is presented in Table 1.

**Table 1:** Macronutrient composition, energy density and calories from macronutrients.

Macronutrients		Regular Chow (7012 Teklad LM-485)	KD (TD.96355)
Protein	%	19.1	15.3
Carbohydrate	%	44.3	0.5
Fat	%	5.8	67.4
Fiber	%	4.6	8.8

Energy density	kcal/g	3.1	6.7

Calories from:			
Protein	%	25	9.2
Carbohydrate	%	58	0.3
Fat	%	17	90.5

### 2.3 Animal anesthesia and preparation

Mice were anesthetized during electrophysiological recordings and during the muscle contractility assessment via inhaled isoflurane delivered at 3–5% for induction and 1–2% for maintenance anesthesia using a SomnoSuite low-flow anesthesia system (Kent Scientific, Torrington, CT). The body temperature was maintained at 37 °C with an infrared heating pad (Kent Scientific). To avoid corneal dryness, a petroleum-based eye lubricant (Dechra, Northwich, UK) was applied. Hair of the right hindlimb was shaved (model VPG 6530, Remington, DeForest, WI). The electrophysiology and contractile procedures were carried out as we have described below [31,33,34].

### 2.4 Grip strength test

Bilateral hindlimb and all limb grip strength were assessed as previously described using a force transducer (Model GT3, Bioseb SAS BP32025-F-13845 Vitrolles Cedex, Pinellas Park, FL, USA) [35]. Mice were grasped and allowed to grip a T-shaped bar connected to the transducer and then were pulled away from the grip meter using a steady and constant motion until grip was lost. For all limb grip, mice were allowed to grip a grid connected to the force transducer and were pulled away from the grip meter using a steady and constant motion until grip was lost. Three trials of hindlimb and all limb grip strength were completed, and the average of the three trials (in grams) was used for analyses. To ensure accuracy and reduce fatigue-related variability, we maintained a rest period of 2 min between each trial. This interval was carefully chosen based on established research practices and prior studies in the field, as exemplified by Ari et al. (2020), who successfully employed a similar approach in their investigations of motor performance [36]. By providing a sufficient rest period between trials, we aimed to minimize the potential for muscle fatigue, which could otherwise influence the test results. The average of the three trials was then calculated to obtain a representative measure of each rodent's grip strength.

### 2.5 Rotarod latency to fall

Coordination and motor performance were analyzed and conducted using the rotarod test (BX-ROD, Bioseb). To start the test, once the mice were placed on the rod, the rod started rotating at 4 rpm and accelerated at 1 rpm/6 s to a maximum of 40 rpm [37]. Three trials were performed at each timepoint and averaged. For our study, consistent with Barreto et al. (2010), we used the maximum latency to fall by determining the time in which the mouse was able to remain on the rotarod before falling for a maximum duration [38].

### 2.6 Electrophysiological Assessment of Motor Unit Size and Number and Neuromuscular Junction Transmission

Motor Unit Number Estimation (MUNE) was performed to estimate the number of functioning motor units using an approach similar to previous studies in aged mice via a clinical electrodiagnostic system (Cadwell, Kennewick, WA, USA) [31,33]. A pair of 28-gauge insulated monopolar needle electrodes (Teca, Oxford Instruments Medical, NY, USA) were inserted subcutaneously into the proximal hindlimb in the region of the sciatic nerve as the cathode and anode for stimulation. A pair of fine wire ring electrodes (Alpine Biomed, Skovlunde, Denmark) were used as the active electrode (placed over proximal gastrocnemius just distal to the knee) and reference electrode (placed over the metatarsal area of the right foot). The ground electrode was placed on the tail (Care Fusion, Middleton, WI, USA). Low frequency and high frequency filters were set at 10 Hz to 10 kHz, respectively. To determine MUNE, first, the peak-to-peak amplitude of the compound muscle action potential was recorded following supramaximal stimulation of the sciatic nerve (constant current: <10 mA, duration 0.1 ms). Then, 10 incremental, all-or-none responses obtained during a gradually increasing stimulations were recorded and averaged to calculate the average single motor unit potential (SMUP) amplitude. Then, MUNE was calculated as such: MUNE = Peak-to-peak CMAP Amplitude/ SMUP.

Repetitive Nerve Stimulation (RNS) testing was then performed using the same recording setup as described for MUNE. During RNS, trains of 10 stimulations were delivered at 50 Hz. We used 50 Hz stimulation in the present study guided by its established use in preclinical models, as well as its relative technical feasibility and reliability. For instance, the work of Padilla et al. (2021) effectively demonstrates the utility of this approach in profiling age-related muscle weakness and wasting, with a specific focus on neuromuscular junction transmission [39]. Amplitude changes between the first and 10th stimulations were calculated using the following formula: % amplitude decrease = [(Amplitude of 10th response − Amplitude 1st response)/Amplitude of 1st response] * 100%.

### 2.7 Plantar flexion muscle contractility

For muscle contractility testing, mice were placed in supine on the testing platform to assess the right hindlimb using an in vivo muscle contractility system (Aurora Scientific Inc, Canada Model 1300A Muscle). The right hindlimb was taped to a rotating foot plate connected to a dual control motor to assess plantar flexion torque. Then, the hindlimb was locked into testing frame connected to the platform base using blunt clamps at the femoral condyles taking care to avoid injury of the fibular nerve at the fibular head. The tibial nerve located in the posterior medial knee was then stimulated using two insulated monopolar electrodes placed subcutaneously (Natus Neurology Inc, Middleton, WI, USA). Stimulation was adjusted (constant current: 0−10 mA, 0.2 ms) to determine the intensity required for a maximal twitch response and adjusted to 120–150% to ensure maximal stimulation. Peak twitch torque was measured after a single 0.2 ms supramaximal pulse-wave stimulation. Tetanic torque was measured using a 200 ms train of stimuli delivered at 125 Hz. This entire process was carried out as previously described [31,33,40].

### 2.8 Blood collection

Blood beta-hydroxybutyrate and blood glucose were assessed using the Keto mojo B Ketone and Blood Glucose Monitoring System Test Kit. Mice were not fasted at any time during the study. This decision was based on the objective of maintaining a consistent and stress-free environment for the animals, as fasting could introduce additional variables that might affect their physiological responses. To ensure uniformity in the collection process and minimize variability in the results, blood samples were collected from the submandibular area of all rodents at a consistent time of 8:00am for both the KD and regular chow groups. This timing was strategically chosen to align with the animals' regular circadian rhythms and feeding patterns, thereby providing a representative snapshot of their physiological state under normal living conditions. By collecting blood samples at the same time each day, we aimed to reduce any diurnal variations that might influence the results. The mouse was held in one hand using the index finger and thumb which applied the desired pressure to the maxillary vein. The maxillary vein was located along the curvature of the mandible, just below this mark in the groove that runs through the mandible. Using a lancet (needle), firm pressure was applied to the maxillary point, caudal to the eye and ventral to the ear, where the submandibular vein is located, then released until blood flowed. The lancet was kept perpendicular to the bleeding site to avoid injuring the ear canal. Ketone and glucose test strips were then placed under the puncture site using the Keto – Mojo GK + blood glucose & B – ketone dual monitoring system instrument until the desired volume of blood was collected for measurement in mmol/L. Finally, gentle pressure was applied with a gauze over the mandibular area to stop the bleeding of the mouse.

### 2.9 Wet weight

At the end of the study, the mice were deeply anesthetized before being sacrificed. The gastrocnemius and soleus muscles of the right hindlimb were dissected and removed. Wet weight in grams was recorded on a previously calibrated scale.

### 2.10 Statistical analyses

Statistical analyzes were performed using the GraphPad Prism 10.1.2 program (GraphPad Software Inc., San Diego, CA, USA). To identify whether there was a trend for an effect of the intervention on body mass, the Eta^2^ effect size (ƞ^2^) was used. For the grip test, rotarod performance test, electrophysiology and muscle contractility analyses, two-way ANOVA mixed-effects analysis (diet and time × diet) was used to compare the dietary groups across time points. To determine group differences in blood ketone and glucose levels in the intervention group and control group, Mean and SD, Eta^2^ effect size (ƞ^2^), and unpaired t-test were used. Statistical significance was set at p < 0.05.

## 3 Results

### 3.1 KD significantly increases blood ketones but shows minimal impact on body mass

**Fig. 2:**
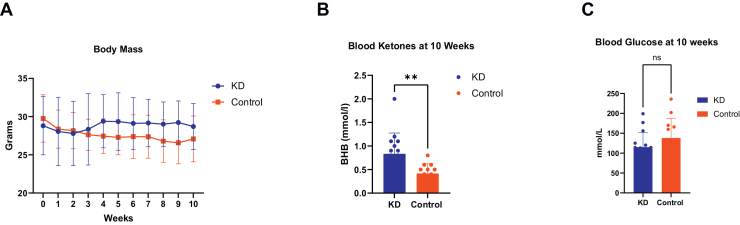
Body mass, beta-hydroxybutyrate and glucose levels. (A) Body mass showed a moderate effect for increasing in the ketogenic diet (KD) group relative to the control group (η^2^ = 0.422), but this effect did not reach statistical significance (Mixed-effects analysis two-way ANOVA time × diet, p = 0.0882). (B) Beta-hydroxybutyrate (BHB) was significantly higher at 10 weeks in mice on a KD vs control group (0.8333 ± 0.44 mmol/l versus 0.42 ± 0.21 mmol/l, η^2^ = 0.265, unpaired t-test, p = 0.0060). (C) Blood glucose at 10 weeks showed a moderate reduction in the ketogenic diet group versus control group, but this effect did not reach statistical significance (115.1 ± 37.11 mmol/L/ versus 138 ± 49.11 mmol/L, η^2^ = 0.070, unpaired t-test, p = 0.1800). **p < 0.01.

Body mass in C57BL/6 mice treated with a KD did not demonstrate a statistically significant increase in comparison to the control group although a moderate effect size was observed (η^2^ = 0.422) (Fig. 2A). As expected, mice treated with a KD showed a 2-fold greater level of BHB at 10 weeks (p = 0.0060) (Fig. 2B). In addition, the group of mice treated with a KD showed a slight but not significant reduction in blood glucose at 10 weeks of study (η^2^ = 0.070).

### 3.2 Motor function and motor unit integrity are improved in aged mice fed a ketogenic diet

**Fig. 3:**
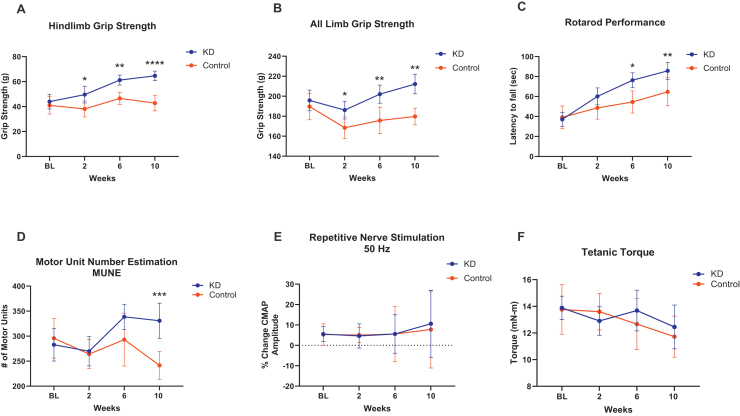
Aged mice fed on a ketogenic diet show improved motor function and motor unit number. Mice in the KD group demonstrated significance increases in (A) Hindlimb grip strength (Mixed-effects analysis two-way ANOVA diet, p = 0.0001, time × diet, p = 0.0030); (B) All limb grip (Mixed-effects analysis two-way ANOV diet, p = 0.0005, time × diet, p = 0.0523) and (C) Rotarod latency to fall (Mixed-effects analysis two-way ANOVA diet, p = 0.0126, time × diet, p = 0.0021). Mice in the KD group also demonstrated significance increase in (D) Motor unit number estimation (MUNE) (Mixed-effects analysis two-way ANOVA diet, p = 0.0465, time × diet, p = 0.0064) but not in (E) peak tetanic muscle contractility torque at 150 Hz stimulation at baseline to 10 weeks (Mixed-effects analysis two-way ANOVA diet, p = 0.5248; time × diet, p = 0.5836) or (F) repetitive nerve stimulation at 50 Hz recorded at baseline to 10 weeks (Mixed-effects analysis two-way ANOVA significant diet, p = 0.3562; time × diet, p = 0.9871). Plot values are mean with 95% CI. Šídák's multiple comparisons test adjust p values: *p < 0.05, **p < 0.01, ***p < 0.001, ****p < 0.0001. BL = baseline.

Both hindlimb and all limb grip strength were significantly improved in mice treated with a KD compared with a control group (diet, p = 0.0001−0.0005; time × diet p = 0.0030−0.0523) (Fig. 3A and B). Specifically, at endpoint the hindlimb grip showed a 36% increase in the KD group versus 3% in the control group, and all-limb grip showed a 6% increase in the KD group versus 5% decrease in the control group. Similarly, rotarod, a measure of motor stamina and coordination, was improved in mice on the KD showing of 78% increase over the 10-weeks study versus 48% increase in the control group (diet, p = 0.0126; time × diet, p = 0.0021) (Fig. 3C).

MUNE is an electrophysiological technique that can be used to monitor the functional status of a motor unit pool in vivo [11]. MUNE was higher in mice of the KD group showing a 16% increase at 10 weeks of study versus a -20% reduction in the control group (diet, p = 0.0465; time × diet, p = 0.0064) (Fig. 3D). RNS, a measure of neuromuscular junction transmission, did not exhibit a significant result in diet intervention (p = 0.3562), and time × diet interaction (p = 0.9871) (Fig. 3E).

### 3.3 No differences for muscle contractility with KD intervention

In contrast to measures of motor and motor unit function, the changes in muscle contractility were similar between aged mice in the KD and regular chow groups (Mixed-effects analysis two-way ANOVA diet, p = 0.5248; time × diet, p = 0.5836) (Fig. 3E).

### 3.4 Ketogenic diet did not show effects on the wet weight of gastrocnemius and soleus

**Fig. 4:**
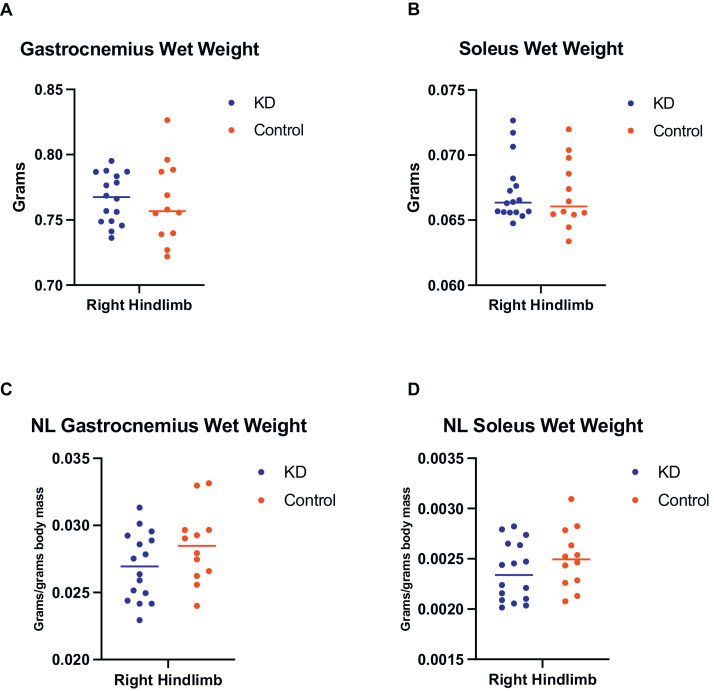
Ketogenic diet on gastrocnemius and soleus wet weight and normalized (NL) in aged C57BL/6 mice. No significant changes in gastrocnemius and soleus wet weight (whole) and normalized by body mass were observed after consumption of the KD ad libitum for 10 weeks. (A) Mean ± SD for gastrocnemius wet weight in the KD group was 0.7621 ± 0.0190 and for the control group 0.7636 ± 0.0310 (η^2^ = 0.003, unpaired t-test, p = 0.7621). (B) For the soleus wet weight in the KD group it was 0.0672 ± 0.0024 and for the control group 0.0670 ± 0.0026 (η^2^ = 0.001, unpaired t-test, p = 0.8497). (C) Mean ± SD for NL gastrocnemius wet weight in the KD group was 0.0269 ± 0.0025 and for the control group 0.0284 ± 0.0027 (η^2^ = 0.080, unpaired t-test, p = 0.1434). (D) NL soleus wet weight in the KD group it was 0.0023 ± 0.0002 and for the control group 0.0025 ± 0.0002 (η^2^ = 0.052, unpaired t-test, p = 0.2402).

The ketogenic diet intervention was evaluated for 10 weeks in C57BL/6 mice at 25 months of age on the wet weight of the gastrocnemius (p = 0.7621, η^2^ = 0.003), and soleus (p = 0.8497, η^2^ = 0.001) muscles on the right side of the hindlimb, with no group differences observed in the results (Fig. 4A and B). Similarly, no significant differences were observed in muscles normalized by body mass, gastrocnemius, p = 0.1434, η^2^ = 0.080 and soleus, p = 0.2402, η^2^ = 0.052 (Fig. 4C and D). The rationale for normalizing wet muscle weight against body mass is to mitigate the potential biases that may arise due to weight variability among the study rodents during the course of the study. This normalization process is crucial for ensuring that the grip strength measurements are not unduly influenced by the individual size or weight of the mice. By accounting for these variables, we can more accurately assess the true muscular strength and functionality, independent of body size. This approach enhances the comparability of data across different subjects and ensures that any observed differences or similarities in grip strength are reflective of muscle performance rather than body weight variations.

## 4 Discussion

In the present study, we examined the effect of 10 weeks of a KD on motor function and indices of sarcopenia in aged C57BL/6 mice. There is building evidence that integrity of the nervous system is a major contributor to sarcopenia in older adults [41]. Despite this, few interventions, including nutritional, have been investigated for improving sarcopenia through neurological mechanisms. It was previously reported that the KD increases lifespan in male mice by 13.6% and preserved physical function [22]. Our study adds to this body of knowledge as it demonstrates that a KD intervention improves motor function and motor unit function in aged mice.

### 4.1 Ketogenic diet intervention not manifested significant changes in body mass

The primary function of a ketogenic (high-fat) diet is to shift the body's metabolism from relying primarily on carbohydrates to utilizing fats as the main energy source. This metabolic adaptation can often lead to a stabilization of body mass, rather than the significant weight loss or gain typically associated with dietary changes [42,43]. This phenomenon is documented in several studies, including the work of Kennedy et al. (2007) and Weber et al. (2022), which have explored the unique metabolic states induced by KD in mice [42,43]. Our findings, in conjunction with existing literature, suggest that the KD’s role in metabolic adaptation and energy utilization in mice may not manifest as significant changes in body mass. This insight contributes to our understanding of the complex effects of KD in rodent models.

### 4.2 Ketogenic diet improved muscle strength and motor performance during aging in mice

Our results show that the 10-week KD intervention significantly and progressively improved hindlimb and all-limb grip strength. Similar results were found by Roberts et al. (2017) where, after a KD intervention, aged mice showed greater grip strength compared to those in the control group [22]. Other studies by Ahola-Erkkila et al. (2010) also showed an effect of the KD in the context of other forms of muscle weakness including a mouse model of late-onset mitochondrial myopathy [44]. It is important to note that this prior work indicated that the KD intervention enhances muscle strength even in the absence of a physical/exercise stimulus. For example, in a study by Camajani et al. (2022) using adults that were 50–70 years of age who undertook a KD without physical/exercise training showed a significant increase in muscle strength and function [45]. These results demonstrate the potential of the KD intervention to improve muscle strength during aging. Another study carried out by Zhou et al. (2023) also demonstrated the ability of the KD on forelimb muscle strength under isometric contraction in the grid-wire suspension test during aging in male mice [46]. It has been suggested that the reason a KD enhances muscle function in the context of aging is due to the availability of ketone bodies (BHB), which provide energy substrates [22,29]. On the other hand, the improvement in strength in our study might be attributed to enhanced central neural activation rather than direct peripheral effects. This is supported by literature illustrating the impact of the KD on cortical functions, notably its established use in managing epilepsy. For instance, the study of Cantello et al. (2007) suggests that the KD can significantly influence central nervous system (CNS) functioning [47]. This underscores the potential of the KD not just as a therapeutic diet for metabolic and neurological disorders but also in modulating CNS activities, which could explain the observed improvements in muscle strength and motor performance in aging mice.

In our study, mice fed with the KD showed significant increase in the motor performance on grip strength testing and latency to fall during rotarod testing. Beckett et al. (2013) previously demonstrated that APP/PS1 knock-in mice fed KD ad libitum for one month (endpoint 2–3 months old) showed better rotarod performance [48]. Other work has shown that the KD can improve motor performance, and that exogenous ketone supplementation in rodents may be a viable alternative for those who cannot consume a KD [49]. For instance, a study using a 5-month-old mouse model of Alzheimer's disease fed for 16 weeks on the KD showed that the average latency to fall in the accelerating rotarod was significantly higher in the KD-fed mice compared to the control group [50]. We acknowledge that these improvements could be partially attributed to a training (motor learning) effect, as the mice become more adept at the task with repeated trials. This phenomenon is a common consideration in longitudinal studies involving motor skills. Furthermore, there is a possibility that the KD group's enhanced performance on the rotarod test – indicated by their increased latency to fall – might not be solely due to physical improvements. KD may also have a cognitive component, potentially enhancing the mice's ability to learn and adapt to the task more efficiently. This suggestion is grounded in the understanding that dietary interventions, particularly those like the KD known for their neurological impacts, can influence cognitive functions including learning and memory. Therefore, while the improvements in rotarod performance can be seen as an indication of enhanced motor abilities, it is also plausible that these improvements reflect a synergistic effect of physical training and enhanced cognitive function due to the KD. The diet intervention may not only improve the metabolic efficiency of the muscles involved but also contribute to a more rapid and effective learning process in the diet-induced test.

As a part of this learning process, Kim et al. (2021) has reported that ketones can improve learning in multiple rodent models, suggesting a potential mechanism for the increased motor performance observed in our non-pathological mouse models [51]. The KD impact on cognitive functions might indirectly enhance motor performance through improved learning and coordination. Additionally, Ari et al. (2020) and Zou et al. (2016) have shown that ketones may enhance muscle performance and CNS control of muscle contractions, contributing to improved motor abilities in rodents [36,52]. This effect is observed not only in models with motor dysfunction but also in normal rodents, indicating that the benefits of KD may extend beyond pathological conditions. While these findings from models of cognitive impairment and muscle dysfunction offer important context, we recognize that they should be interpreted with caution when applied to our study. The mechanisms underlying the KD's effects in these models may differ due to the distinct metabolic and neurological pathways involved in pathological conditions.

Nevertheless, these studies provide a broader perspective on the KD potential effects, suggesting that it could have multifaceted benefits on motor performance, muscle health, and cognitive function. For example, Pathak et al. (2022) demonstrated that a two-month KD intervention in middle-aged female mice resulted in notable cognitive improvements, particularly in behaviors associated with anxiety, memory, and exploration [53]. These cognitive enhancements were linked to biochemical changes, such as increased levels of PGC1α protein in the gastrocnemius muscle and a tissue-specific rise in mitochondrial mass and kynurenine aminotransferase (KAT) levels. The KAT proteins, by converting kynurenine to kynurenic acid, suggest a potential muscle-brain communication pathway in KD mice that could underlie the observed cognitive benefits. Interestingly, Pathak et al. (2022) found that, despite these cognitive and biochemical improvements, motor functions such as grip strength and coordination were not significantly impacted by the ketogenic diet in the short term [53]. This finding introduces a nuanced perspective on the diet's effects, highlighting the possibility that its neurocognitive benefits may be more pronounced or occur earlier than changes in motor function. In contrast, our study observed more significant motor function improvements, particularly in the later stages (week 10 as opposed to week 6). This disparity suggests that the impact of the KD on motor functions may be more pronounced over a longer duration. It raises the possibility that while short-term interventions might predominantly influence cognitive aspects, prolonged exposure to the diet could lead to gradual improvements in motor function. This comparison underscores the complex and multifaceted nature of the KD effects. It indicates that the time frame of the diet intervention and the specific outcomes measured (cognitive vs. motor function) can influence the observed benefits. Our findings, in conjunction with the study by Pathak et al., suggest that KD has the potential to impact both cognitive and motor functions, but the extent and timeline of these effects may vary. In summary, these results suggest that the KD could play an important role in improving cognitive and motor function in aging mice.

### 4.3 Motor unit number was higher in aged mice fed a ketogenic diet

The number of functional motor units that innervate a muscle is a fundamental element for neuromuscular control, and losses of motor units is considered a potentially important factor in the development of sarcopenia [31,54,55]. Prior work has shown that estimates of motor unit numbers using MUNE are reduced in older adults [[56], [57], [58]]. Furthermore, there is an observed progression in motor unit loss with age, coupled with maladaptive changes in terminal Schwann cells, leading to reduced capacity for compensatory reinnervation in advanced ages. Our findings suggest an increase in the number of motor units in mice on a KD during week 6, with maintenance of these levels until week 10. This pattern contrasts with the control group, where a reduction in motor units was noted over the same period. Similar to our findings in wild-type mice, a study by Zhao et al. (2006) reported that KD administration resulted in increased motor neuron survival and improved motor function in a G93A-SOD1 transgenic mouse model. This could be relevant to our study as it could explain how the KD helps to improve and increase the motor units’ number that are lost during aging. The results from Zhao et al. (2006) are supported by the fact that the KD induced elevation of blood ketones could ameliorate mitochondrial defects by increasing mitochondrial function and ATP production [59]. The results of Zhao and our study suggest that the KD intervention may improve muscle fiber innervation of an alpha motor neuron, triggering better function of the neuromuscular system during aging. Based on our study results the physiological effect of the KD on MUNE may have been the result of increased plasma ketone bodies, especially BHB, the most abundant ketone found in circulation. Based on prior work, increase of ketones bodies may improve mitochondrial respiration, promote long-term neuronal potentiation, increase expression of brain-derived neurotrophic factor, increase G-coupled protein receptor signaling, attenuate oxidative stress, reduce inflammation, and alter post-translational modifications of the protein through lysine acetylation and BHB [25].

More studies are needed to clearly establish the mechanisms of the KD on the number of motor units in advanced ages. KD can cause some molecular changes to potentiate neuronal plasticity [60]. One possibility is that KD induced plasticity may promote improved innervation and thus maintenance of MUNE during aging. For example, KD increases serum leptin levels as early as 5 days after starting the diet [61] and leptin is known to be not only neuroprotective but also to enhance neuronal plasticity in vitro and in vivo [62,63]. Additionally, the stimulatory effect of BHB on mitochondrial density could facilitate neuronal plasticity. Dendritic mitochondria have been implicated in synapse formation and the number of mitochondria correlates with the number of newly formed synapses [[64], [65], [66]]. Another possible explanation for the maintenance of MUNE with the KD during aging is that the induction of ketone metabolism may influence synaptic morphology and function, as suggested by Veyrat-Durebex et al. (2018) [67]. This includes potential modifications in ion channels, glutamatergic transmission, and synaptic vesicular cycle machinery. Such changes could potentially explain the maintenance or slight increase in MUNE observed in our study. The diet's impact on these cellular and molecular pathways might contribute to preserving motor units against the typical decline seen with aging. While our findings indicate a potential protective effect of the KD on motor units during aging, we agree that further research is necessary to fully understand these mechanisms. Specifically, exploring the link between diet-induced metabolic changes and neuromuscular health could provide valuable insights.

During aging a decrease in muscle activity occurs, causing weakness and that could be related to reductions in MUNE [68]. Studies by Arasaki et al. (2006) demonstrated that people who did not present muscle weakness also showed no changes in MUNE [68]. Other results show that motor unit losses could be supported by the loss of CMAP and MUNE and that these motor unit losses could be compensated by the reactivation and remodeling of motor unit size [69,70]. To better understand this, we must mention that altered axonal transport, as well as altered neuromuscular transmission and myofibril atrophy, are part of the mechanisms for the loss of motor units [71]. Studies such as that of Kariyawasam et al. (2021) demonstrate that the recovery of the number of motor units is correlated with functional change, which highlights the capacity of MUNE measures to act as a biomarker for therapeutic response [71]. This could be explained based on studies such as that of Li et al. (2020) where they supplied a ketogenic diet for 8 weeks in rats and found an increase in the average myelin thickness and axon/fiber diameter compared to controls as well as an improvement in functional recovery after sciatic nerve injury [72]. Other studies such as that of Liskiewicz et al. (2016) found that regenerating nerves in the preconditioned KD group were more similar to those in uninjured rats based on a variety of histomorphometry parameters, including myelin thickness, fiber density, and fiber diameter [73].

It is important to note that MUNE represents an estimation of the number of “functional” motor units supplying a particular muscle and not necessarily anatomical count. Thus, reactivation of “silent” motor units is one potential explanation of the higher MUNE value observed. Another possible explanation includes an effect of the KD on plasticity of innervation at the neuromuscular junction. There is evidence that aging results in polyneuronal innervation, which would theoretically reduce calculated MUNE [74]. Thus, if KD normalizes innervation to monosynaptic state, MUNE would be increased. The lack of impact of the KD on contractility argues in favor of normalization of polyneuronal to monosynaptic innervation. However, visual examination of the data suggests upward trends of the data at 6 weeks (both controls and KD diet groups) raises the additional possibility of technical variability.

These results could shed light on our study on the therapeutic effect of the ketogenic diet by remodeling the number of motor units for 10 weeks in an aged mouse model. Our study contributes to this emerging area of research by suggesting a possible beneficial role of the KD in maintaining motor unit integrity in aged mice.

### 4.4 Muscle contractility was not improved by the ketogenic diet

The intrinsic force producing capacity of skeletal muscle decreases with advancing age in humans and mice [75]. The soleus muscle of female C57BL/6 mice demonstrates a reduction in maximal tetanic force ∼4, 8-, 16-, 24-, and 28-months during aging but muscle size and contractile protein content were not affected [76]. This may have been the result of the authors being unable to clearly explain the reduction in strength that occurs with aging. Furthermore, it should be noted that preservation of motor function through KD has been associated with higher relative weights in hindlimb muscles in aged mice. However, our study did not show significant increases with the intervention of the KD in gastrocnemius and soleus wet weight during aging. In our study we wanted to examine the effect of the KD (∼90% fat and 10% protein) on muscle contractility during aging in C57BL/6 mice. The results show that tetanic torque was not affected by the KD intervention for 10 weeks. Similarly, previous studies showed that feeding the KD for 4 weeks did not affect tetanic contraction in aged male rats [77]. According to the results of our study, the KD increased hindlimb and all limb muscle strength but did not affect gastrocnemius muscle contractility during aging. These results could support the idea that the KD intervention improves central neural activation without a direct peripheral impact. This perspective is supported by the work of Pietrzak et al. (2022), who have explored the therapeutic role of the KD in neurological disorders [78]. We acknowledge that the effect of KD on MUNE indicates a potential impact on neuromuscular connectivity. However, as pointed out, the presence of a learning effect from serial testing cannot be ruled out. This effect could contribute to improved performance in tasks such as the rotarod test, independent of direct neurological or muscular changes. Therefore, while our findings suggest a potential role for KD in enhancing motor function, we argue that more research is needed to fully understand the underlying mechanisms. It is premature to conclude definitively that the observed improvements are solely due to neurological changes without considering the multifactorial nature of motor function, which encompasses both neural and muscular components.

By adopting this stance, we aim to emphasize the need for further studies to dissect the intricate interplay between diet, neurological function, and muscle performance. Our current findings provide a foundation for such investigations, highlighting the potential of KD in modulating motor function but also underscoring the complexity of attributing these effects to specific mechanisms.

### 4.5 Ketogenic diet on muscle physiology

There is a potential benefit of KD in muscle physiology in that the KD might influence muscle fiber composition, particularly favoring oxidative fibers (type IIa) over glycolytic fibers (type IIb), as suggested by Nilwik et al. (2013) [79]. This shift could be crucial in understanding age-related muscle size and strength loss. Additionally, the KD could enhance axonal sprouting and fiber bundling, as well as alter fuel utilization towards aerobic respiration and beta-oxidation, potentially preserving oxidative fibers, based on findings by Lin et al. (2014) [80]. These discussions provide an additional theoretical basis for the observed effects of KD in our study, setting the stage for more detailed investigations in the future.

While our study provides compelling evidence of the KDs efficacy in improving motor function and neuromuscular integrity in aged mice, it is crucial to approach the translational potential of these findings to human research with caution. The observed increase in plasma β-hydroxybutyrate (BHB) levels and the subsequent enhancements in motor and neuromuscular function underscore the KD's promise as a nutritional intervention in the context of aging. However, the direct applicability of these results to humans requires careful consideration. Sarcopenia, characterized by the progressive loss of muscle mass and strength, presents a significant challenge in aging human populations. While the KD's role in elevating ketone bodies suggests a potential avenue for mitigating such age-related declines, the complex interplay of metabolic, genetic, lifestyle, and environmental factors in humans necessitates rigorous, controlled clinical trials to validate these effects. Importantly, the extrapolation of findings from mouse models to human conditions should be undertaken with care, acknowledging the differences in metabolism, lifespan, and response to dietary interventions between species. Our study, therefore, highlights the need for further research that specifically addresses these translational gaps, aiming to explore the feasibility, safety, and efficacy of KD as a therapeutic strategy to combat sarcopenia and enhance neuromuscular health in the human aging process.

## 5 Limitations

There are several limitations of this work that should be noted. The first limitation concerns our inability to blind the outcome assessor. This issue stems from the fact that mice fed a high-fat diet demonstrate oiliness in their coats, making blinding impractical. Another limitation relates to our technique for assessing NMJ transmission efficacy. Based on our prior work in aged rats, we chose 50 Hz RNS as our outcome of NMJ transmission [39]. While SFEMG is more sensitive [81], we have concerns that this technique would result in notable muscle damage confounding the study when performed serially in mice due to electrode size relative to muscle size.

There are other limitations that must also be considered. For instance, we used the KD to increase ketone bodies in the study mice (9.2% protein, 0.3% carbohydrates and 90.5% fat), but did not quantify the amount of food or calorie intake ingested by the mice (5 mice per cage). We also did not track ketosis status during the study to examine the relationship between the degree of ketosis and functional outcomes. Additionally, we chose to use the 7012-chow diet instead of a semi-purified chow diet based on its successful application in prior research publications [[82], [83], [84]].

While we used male and female mice the study, it was not powered to permit examination of sex-specific effects. Male and female mice, like humans, not only respond differently to various treatments, but each sex has specific hormones and genes that could affect the results of the investigation. Although research with mice cannot always be duplicated in humans, the use of both sexes of mice in the present study may have led to a better understanding of how the KD affects motor function and the number of motor units during aging. Thus, further work is needed that more fully considers sex as a biological variable.

Another limitation was the absence of histological data in our study, particularly pertaining to muscle tissues. We recognize the importance of histological analysis in providing a more comprehensive understanding of the physiological impacts of KD which we anticipate will yield valuable insights into the cellular and structural changes induced by our dietary interventions. Lastly, future studies examining the differential effects of ketogenic (endogenous), and ketone esters (exogenous) diets are also needed to clearly identify the physiological effects and consequences of each intervention.

## 6 Conclusions

In this study we establish that the KD improves motor function in aged mice and demonstrate that this improved function may be related to improved motor unit connectivity with muscle. We observed that the 10-weeks KD intervention improved muscle strength, motor performance, and increased the number of functioning motor units, but had no effect on muscle contractility, mass, and/or an index of neuromuscular transmission during aging. These results strengthen previous research on the potential of KD therapies to improve motor function during aging and support the potential of a KD to improving or maintaining function in older adults.

## Funding statement

This research was funded by NIA/NIH R01AG067758 andR01AG067758-S1.

## Conflicts of interest

BC Clark reported receiving grants from the National Institutes of Health during the conduct of the study; grants and personal fees from Regeneron Pharmaceuticals; grants from Astellas Global Development Inc, RTI Solutions, NMD Pharma, and OsteoDx Inc; and personal fees from Gerson Lehrman Group; and reported serving as a co-founder and chief of aging research at OsteoDx Inc outside the submitted work. WD Arnold has received grant funding from NMD Pharma and Avidity Biosciences, consulting fees from Avidity Biosciences, NMD Pharma, Dyne Therapeutics, Genentech, Design Therapeutics, Cadent Therapeutics, Catalyst Pharmaceuticals, and from Novartis. JS Volek is a co-founder and stockholder in Virta Health, receives royalties for low-carbohydrate books, and is a scientific advisor for Simply Good Foods and Cook Keto.

## Ethical standards

The experiments performed during this investigation are following current laws of The Ohio State University, Columbus, Ohio. In addition, all the authors involved in this study followed the standards of ethical behavior expected to carry out the study and to submit the manuscript for publication. In the same way, the research work was carried out after the experimental protocols were approved by the appropriate institutional review board and ensured that they complied with the guidelines of the responsible government agency.
